# Cross-national epidemiology of DSM-IV major depressive episode

**DOI:** 10.1186/1741-7015-9-90

**Published:** 2011-07-26

**Authors:** Evelyn Bromet, Laura Helena Andrade, Irving Hwang, Nancy A Sampson, Jordi Alonso, Giovanni de Girolamo, Ron de Graaf, Koen Demyttenaere, Chiyi Hu, Noboru Iwata, Aimee N Karam, Jagdish Kaur, Stanislav Kostyuchenko, Jean-Pierre Lépine, Daphna Levinson, Herbert Matschinger, Maria Elena Medina Mora, Mark Oakley Browne, Jose Posada-Villa, Maria Carmen Viana, David R Williams, Ronald C Kessler

**Affiliations:** 1 Department of Psychiatry, State University of New York at Stony Brook, Putnam Hall - South Campus, Stony Brook, NY 11794-8790, NY, USA; 2 Department & Institute of Psychiatry, University of Sao Paulo, School of Medicine, Sãu Paulo, Brazil; 3 Department of Health Care Policy, Harvard Medical School, Boston, MA; 4 Health Services Research Unit, IMIM (Hospital del Mar Research Institute), Barcelona, Spain and CIBER en Epidemiología y Salud Pública (CIBERESP), Barcelona, Spain; 5 IRCCS Centro S. Giovanni di Dio Fatebenefratelli, Brescia, Italy; 6 Netherlands Institute of Mental Health and Addiction, Utrecht, The Netherlands; 7 Department of Psychiatry, University Hospital Gasthuisberg, Leuven, Belgium; 8 Shenzhen Institute of Mental Health & Shenzhen Kangning Hospital, Shenzhen, China; 9 Department of Clinical Psychology, Hiroshima International University, Higashi-Hiroshima, Japan; 10 Department of Psychiatry and Clinical Psychology, Saint George Hospital University Medical Center, Balamand University Medical School and the Institute for Development, Research, Advocacy and Applied Care (IDRAAC), Beirut, Lebanon; 11 Directorate General of Health Services, New Delhi, India; 12 Ukrainian Psychiatric Association, Kiev, Ukraine; 13 Hôpital Lariboisière Fernand Widal, Assistance Publique Hôpitaux de Paris INSERM U 705, CNRS UMR 7157 University Paris Diderot and Paris Descartes Paris, France; 14 Research & Planning, Mental Health Services Ministry of Health, Jerusalem, Israel; 15 Institute of Social Medicine, Occupational Health and Public Health University of Leipzig, Leipzig, Germany; 16 National Institute of Psychiatry, Mexico City, Mexico; 17 The University of Tasmania Statewide and Clinical Director Dept of Health and Human Services New Town, Tasmania, Australia; 18 Instituto Colombiano del Sistema Nervioso, Bogota D.C., Colombia; 19 Section of Psychiatric Epidemiology, Institute of Psychiatry, School of Medicine, University of São Paulo, São Paulo, Brazil; 20 Department of Society, Human Development and Health, Harvard School of Public Health, Boston, MA, USA

## Abstract

**Background:**

Major depression is one of the leading causes of disability worldwide, yet epidemiologic data are not available for many countries, particularly low- to middle-income countries. In this paper, we present data on the prevalence, impairment and demographic correlates of depression from 18 high and low- to middle-income countries in the World Mental Health Survey Initiative.

**Methods:**

Major depressive episodes (MDE) as defined by the *Diagnostic and Statistical Manual of Mental Disorders*, fourth edition (DMS-IV) were evaluated in face-to-face interviews using the World Health Organization Composite International Diagnostic Interview (CIDI). Data from 18 countries were analyzed in this report (n = 89,037). All countries surveyed representative, population-based samples of adults.

**Results:**

The average lifetime and 12-month prevalence estimates of DSM-IV MDE were 14.6% and 5.5% in the ten high-income and 11.1% and 5.9% in the eight low- to middle-income countries. The average age of onset ascertained retrospectively was 25.7 in the high-income and 24.0 in low- to middle-income countries. Functional impairment was associated with recency of MDE. The female: male ratio was about 2:1. In high-income countries, younger age was associated with higher 12-month prevalence; by contrast, in several low- to middle-income countries, older age was associated with greater likelihood of MDE. The strongest demographic correlate in high-income countries was being separated from a partner, and in low- to middle-income countries, was being divorced or widowed.

**Conclusions:**

MDE is a significant public-health concern across all regions of the world and is strongly linked to social conditions. Future research is needed to investigate the combination of demographic risk factors that are most strongly associated with MDE in the specific countries included in the WMH.

## Background

Major depression is a serious, recurrent disorder linked to diminished role functioning and quality of life, medical morbidity, and mortality [1,2]. The World Health Organization ranks depression as the fourth leading cause of disability worldwide [3], and projects that by 2020, it will be the second leading cause [4]. Although direct information on the prevalence of depression does not exist for most countries, the available data indicate wide variability in the prevalence rates. Weissman *et al*. [5] published the first cross-national comparison of major depression as defined by the *Diagnostic and Statistical Manual of Mental Disorders*, third edition (DSM-III) from 10 population-based surveys that used the Diagnostic Interview Schedule (DIS) [6]. The lifetime prevalence ranged from 1.5% (Taiwan) to 19.0% (Beirut), with the midpoints at 9.2% (West Germany) and 9.6% (Edmonton, Canada). The 12-month prevalence ranged from 0.8% (Taiwan) to 5.8% (Christchurch, New Zealand), with the midpoints at 3.0% (US) and 4.5% (Paris). A subsequent cross-national comparison [7] included 10 population-based studies that used the World Health Organization (WHO) Composite International Diagnostic Interview (CIDI) for the revised third edition and the fourth edition of the DSM (DSM-III-R) and (DSM-IV) [8]. Consistent with the earlier report [5], the lifetime rates ranged from 1.0% (Czech Republic) to 16.9% (US), with midpoints at 8.3% (Canada) and 9.0% (Chile). The 12-month prevalence ranged from 0.3% (Czech Republic) to 10% (USA), with midpoints at 4.5% (Mexico) and 5.2% (West Germany). Most recently, Moussavi *et al*. [9] summarized data on depressive episodes as defined by the International Classification of Diseases, 10th revision (ICD-10) in participants in the WHO World Health Survey used in 60 countries, noting that the 1-year prevalence was 3.2% in participants without comorbid physical disease, and 9.3% to 23.0% in participants with chronic conditions.

The wide variability in lifetime and 12-month prevalence estimates of major depression is presumably due to a combination of substantive (genetic vulnerability and environmental risk factors) and measurement (cultural differences in the acceptance and meaning of items, and the psychometric properties of the instruments) factors. Differences in study design might also be involved. That is, apart from administering a common interview schedule, the surveys were not designed as replications with a standard protocol for translation, interviewer training, sampling and quality control. More recently, the WHO World Mental Health (WMH) Survey Initiative conducted a coordinated series of studies using a common protocol and a common instrument, the WHO CIDI, version 3.0 [10], to assess a set of DSM-IV disorders in countries from every continent [11]. The 12-month prevalence of DSM-IV major depressive episode (MDE) in 18 countries ranged from 2.2% (Japan) to 10.4% (Brazil) [12]. The mid-point across all countries was similar to that in previous surveys (5%), as was the weighted average 12-month prevalence for the ten high-income (5.5%) and eight low- to middle-income (5.9%) countries.

Almost all studies find that gender, age and marital status are associated with depression. Women have a twofold increased risk of MDE compared with men [13], people who are separated or divorced have significantly higher rates of depression than the currently married [5,7], and the rate of depression generally goes down with age [5,7]. This evidence, however, comes primarily from studies conducted in western countries. The sparse data available from low- to middle-income countries suggest that the age pattern is either not monotonic or that the association is reversed, with depression increasing with age [12,14]. Other socioeconomic factors have less consistent relationships with depression in different countries [7].

The current report presents data on the prevalence, age of onset and sociodemographic correlates of MDE in 18 countries participating in the WHO WMH Survey Initiative. As noted earlier, each of the WMH surveys used the CIDI for DSM-IV. The CIDI includes a series of diagnostic stem questions to determine which diagnoses are assessed. Unlike previous reports from the WMH or previous surveys, our study used the screening information for MDE in responses to these diagnostic stem questions to conduct an examination of the screen-positive percentages, and of the conditional lifetime and 12-month prevalence of MDE in respondents who endorsed the diagnostic stem questions. This was carried out to investigate the possibility that cross-national differences in prevalence estimates of MDE are due, at least in part, to differences across countries in the optimal threshold of CIDI symptom scores for detecting clinical cases. If such variation exists, we would expect much smaller cross-national differences in endorsement of diagnostic stem questions (which merely ask respondents if they had episodes of several days of being sad or depressed or losing interest in usual activities), than in diagnoses. If this were the case, we would expect the largest cross-national differences in conditional prevalence estimates of MDE to occur in screened positives. If differential variation of this sort exists, it would provide more reason than currently exists to suspect that cross-national differences in optimal diagnostic thresholds of the CIDI symptom scale lead to biased estimates of cross-national differences in prevalence in the WMH data.

A justification for this line of thinking comes from an earlier cross-national WHO study of major depression in primary-care patients, which found strong similarity in the latent structure of depressive symptoms across 14 different countries in different parts of the world, but also found that countries with the highest prevalence estimates generally reported the lowest impairment associated with depression [15]. The authors concluded from these results that although cross-national differences in the estimated prevalence of depression cannot be attributed to differences in the nature or validity of the concept of a depressive episode, it is possible that DSM criteria may define different levels of depression severity in different countries. Our cross-national comparison of responses to diagnostic stem questions, described in the previous paragraph, was designed to shed some light on this possibility. In addition, we carried out a parallel analysis of cross-national differences in impairment associated with MDE.

Results are organized by distinguishing between countries classified by the World Bank [16] as low- or middle-income versus higher-income countries. This distinction was made based on patterns both in the WMH surveys [10] and in other cross-national epidemiologic surveys [7,9], which raise concerns that MDE prevalence estimates might be artificially lower in low- to middle- than higher-income countries due to methodological differences of the types considered here.

## Methods

### Ethics

Procedures for human subject protection were approved and monitored for compliance by the institutional review boards of each local organization coordinating the survey. Informed consent was obtained before beginning interviews in all countries.

### Sampling and procedure

The WMH surveys are a series of community-based studies conducted throughout the world [11]. This paper included data on MDE from ten high-income countries (Belgium, France, Germany, Israel, Italy, Japan, Netherlands, New Zealand, Spain, United States) and eight low- to middle-income countries (Brazil (São Paulo), Colombia, India (Pondicherry), China (Shenzhen), Lebanon, Mexico, South Africa, Ukraine) based on World Bank development criteria [16]. As noted in the introduction, we distinguished results from low- to middle-income versus higher-income countries based on the suspicion that optimal thresholds for defining clinically significant depression might be lower than the CIDI thresholds in the former countries, resulting in underestimation of the prevalence of MDE in the CIDI in those countries. The surveys involved either national household samples or representative samples of urban areas (Table 1). Weights were used to adjust for differential probabilities of selection into the study, and to match the sample sociodemographic distributions with the population distributions within each country. Sample sizes ranged from 2,372 (the Netherlands) to 12,790 (New Zealand), giving a total of 89,037. The average weighted response rate was 71.7% (Table 1).

**Table 1:** World Mental Health (WMH) Survey sample characteristics

					Sample size			
Country	Survey^a^	Sample characteristics^b^	Field dates	Age range	Part I	Part II	Part II and age ≤ 44 years^d^	Response rate^c^
**I. High-income**								
Belgium	ESEMeD	Stratified multistage clustered probability sample of individuals residing in households from the national register of Belgium residents. NR	2001-2	18+	2419	1043	486	50.6
France	ESEMeD	Stratified multistage clustered sample of working telephone numbers merged with a reverse directory (for listed numbers). Initial recruitment was by telephone, with supplemental in-person recruitment in households with listed numbers. NR	2001-2	18+	2894	1436	727	45.9
Germany	ESEMeD	Stratified multistage clustered probability sample of individuals from community resident registries. NR	2002-3	18+	3555	1323	621	57.8
Israel	NHS	Stratified multistage clustered area probability sample of individuals from a national resident register. NR	2002-4	21+	4859	4859	--	72.6
Italy	ESEMeD	Stratified multistage clustered probability sample of individuals from municipality resident registries. NR	2001-2	18+	4712	1779	853	71.3
Japan	WMHJ 2002-2006	Unclustered two-stage probability sample of individuals residing in households in nine metropolitan areas (Fukiage, Higashi-ichiki, Ichiki, Kushikino, Nagasaki, Okayama, Sano, Tamano, Tendo Tochigi)	2002-6	20+	3416	1305	425	59.2
Netherlands	ESEMeD	Stratified multistage clustered probability sample of individuals residing in households that are listed in municipal postal registries. NR	2002-3	18+	2372	1094	516	56.4
New Zealand^**e**^	NZMHS	Stratified multistage clustered area probability sample of household residents. NR	2004-5	18+	12790	7312	4119	73.3
Spain	ESEMeD	Stratified multistage clustered area probability sample of household residents. NR	2001-2	18+	5473	2121	960	78.6
United States	NCS-R	Stratified multistage clustered area probability sample of household residents. NR	2002-3	18+	9282	5692	3197	70.9
**II. Low- to middle-income**								
Brazil	São Paulo megacity	Stratified multistage clustered area probability sample of household residents in the São Paulo metropolitan area, Brazil	2004-7	18+	5037	2942	--	77.7
Colombia	NSMH	Stratified multistage clustered area probability sample of household residents in all urban areas of the country (approximately 73% of the total national population)	2003	18-65	4426	2381	1731	87.7
India	WMHI	Stratified multistage clustered area probability sample of household residents in Pondicherry region, India. NR	2003-5	18+	2992	1373	642	98.6
Lebanon	LEBANON	Stratified multistage clustered area probability sample of household residents. NR	2002-3	18+	2857	1031	595	70.0
Mexico	M-NCS	Stratified multistage clustered area probability sample of household residents in all urban areas of the country (approximately 75% of the total national population)	2001-2	18-65	5782	2362	1736	76.6
South Africa	SASH	Stratified multistage clustered area probability sample of household residents. NR	2003-4	18+	4315	4315	--	87.1
Ukraine	CMDPSD	Stratified multistage clustered area probability sample of household residents. NR	2002	18+	4724	1719	540	78.3
PRC	Shenzhen	Stratified multistage clustered area probability sample of household residents and temporary residents in the Shenzhen area, China	2006-7	18+	7132	2475	1994	80.0
								

^a^ESEMeD (The European Study Of The Epidemiology Of Mental Disorders); NHS (Israel National Health Survey); WMHJ 2002-2006 (World Mental Health Japan Survey); NZMHS (New Zealand Mental Health Survey); NCS-R (The USA National Comorbidity Survey Replication); NSMH (The Colombian National Study of Mental Health); WMHI (World Mental Health India); LEBANON (Lebanese Evaluation of the Burden of Ailments and Needs of the Nation); M-NCS (The Mexico National Comorbidity Survey); SASH (South Africa Stress and Health Study); CMDPSD (Comorbid Mental Disorders during Periods of Social Disruption)

^b^Most WMH surveys are based on stratified multistage clustered area probability household samples in which samples of areas equivalent to counties or municipalities in the USA were selected in the first stage, followed by one or more subsequent stages of geographic sampling (for example,, towns within counties, blocks within towns, households within blocks) to arrive at a sample of households, in each of which a listing of household members was created, and one or two people were selected from this listing to be interviewed. No substitution was allowed when the originally sampled household resident could not be interviewed. These household samples were selected from Census area data in all countries other than France (for which telephone directories were used to select households) and the Netherlands (for which postal registries were used to select households). Several WMH surveys (Belgium, Germany, Italy) used municipal resident registries to select respondents without listing households. The Japanese sample is the only totally unclustered sample, with households randomly selected in each of the four sample areas, and one random respondent selected in each sample household. Fourteen surveys are based on nationally representative (NR) household samples, and two others (Colombia, Mexico) were based on nationally representative household samples in urbanized areas. The Israeli survey is a representative sample of individuals.

^c^The response rate was calculated as the ratio of the number of households in which an interview was completed to the number of households originally sampled, excluding from the denominator households known not to be eligible either because they were vacant at the time of initial contact or because the residents were unable to speak the designated languages of the survey. The weighted average response rate for all countries included was 71.7%.

^d^Brazil, Israel and South Africa did not have an age-restricted part II sample. All other countries, with the exception of India and Ukraine (which were age-restricted to ≤ 39 years) were age-restricted to ≤ 44 tears.

^e^The New Zealand response rate was calculated on the entire survey sample size which was of respondents age 16+ years, giving a total of 12,992. For purposes of this analysis we only used respondents aged 18+ years.

The WMH interviews were administered face-to-face by trained lay interviewers. To reduce respondent burden, the interview was divided into two parts. All respondents completed part I, which assessed a set of core mental disorders, including MDE. Part II assessed additional disorders and correlates, and was administered to all part I respondents who met criteria for a part I disorder, plus a probability subsample of other part I respondents. Part II responses were weighted by the inverse of their probability of selection into part II to adjust for differential selection. Details about WMH survey methods and weighting procedures are presented elsewhere [11,17].

Standardized procedures for interviewer training, translation of study materials and quality control were consistently used in each country [11].

### Measures

#### MDE

Near the start of the interview, the CIDI includes three screening questions about sadness/depressed mood, feelings of discouragement, and loss of interest lasting several days or longer. Respondents endorsing one or more of these questions (screen-positives) were given the remainder of the MDE module. DSM-IV MDE requires the presence of five of nine cardinal symptoms that persist for 2 weeks or longer, are present for most of the day nearly every day, and cause significant distress or impairment. These symptoms are depressed mood and markedly diminished interest or pleasure (one of these must be present to meet the criteria for diagnosis), and clinically significant weight gain/loss or appetite disturbance, insomnia or hypersomnia, psychomotor agitation or retardation, fatigue or loss of energy, feelings of worthlessness or excessive guilt, diminished ability to concentrate or think clearly, and recurrent thoughts of death or suicide. MDE was defined for purposes of the present report without organic exclusions and without diagnostic hierarchy rules [12]. Clinical reappraisal studies conducted in several countries found good agreement between diagnoses of MDE based on the CIDI and independent diagnoses based on blinded reappraisal interviews carried out by a clinician [18].

It is noteworthy that the CIDI interview translation, back-translation and harmonization protocol required culturally competent bilingual clinicians in the participating countries to review, modify and approve the key phrases used to describe symptoms of all disorders assessed in the survey [19]. That meant that the terms used to describe core symptoms of depression (that is, sadness, depression, loss of interest) were customized when the original CIDI wording did not match the terms used in the local settings. However, no attempt was made to go beyond the DSM-IV criteria to develop distinct criteria for depression-equivalents that might be unique to specific countries. It is conceivable that the latter kind of expansion would have led to a reduction in cross-national variation in prevalence estimates. However, as noted in the introduction, previous research has shown that the latent structure of the symptoms of major depression is consistent across countries [15], providing a principled basis for focusing on this criterion set in our analysis.

#### Global impairment

A modified version of the WHO Disability Assessment Schedule-II (WHO-DAS-II) was used to assess frequency and intensity of restrictions in performing normal activities during the 30 days prior to the interview [20]. The activity areas included the number of days the person was unable to carry out their normal daily activities because of problems with physical or emotional health as well as various difficulties in role performance during the days in role. WHO-DAS scores are coded in the range 0 to 100, where 0 represents no impairment and 100 total impairment. Reported levels of impairment were low in all countries, with means in the range 1.0 to 5.5 in high-income countries and 1.1 to 4.8 in low- to middle-income countries.

#### Demographic factors

We examined gender, age (18 to 34, 35 to 49, 50 to 64, 65+), current marital status (separated, divorced, widowed, never married, currently married), living arrangement (alone, with others but not spouse/partner, with spouse/partner), income (low, low average, high average, and high, which were based on country-specific quartiles of gross household earnings in the past 12 months [21]) and education (low, low average, high average or high, which were based on country-specific quartiles that take into consideration the fact that distributions of educational attainment vary widely between countries [22]).

#### Statistical analysis

Cross-tabulations were used to estimate the absolute and relative lifetime and 12-month prevalence of endorsing diagnostic stem questions and meeting DSM-IV/CIDI criteria for a diagnosis of MDE. *F*-tests (linear regression) were used to study differences in global impairment by recency of MDE (past 30 days, past month but not in the past 30 days, prior to the past year, never). Logistic regression analysis was used to examine sociodemographic correlates. Unadjusted odds ratios and 95% confidence intervals are presented for these associations. Because the data were weighted and clustered, the Taylor series linearization method [23] implemented in the SUDAAN software package [24] was used to estimate design-based standard errors. Statistical significance was consistently evaluated using two-sided tests, with *P *< 0.05 considered significant.

## Results

### Prevalence of MDE

On average, about half of the respondents in both high-income (52.3%) and low- to middle-income (54.1%) countries endorsed at least one depression diagnostic stem question (screen-positive). (Table 2) However, the screen-positive rate ranged widely, from < 30% in Japan and Pondicherry (India) to ≥ 60% in France, New Zealand, the USA, Brazil and Ukraine. The ratio of the highest to lowest screen-positive rates across countries was 3.3. On average, the estimated lifetime prevalence was higher in high-income (14.6%) than low- to middle-income (11.1%) countries (*t *= 5.7, *P *< 0.001). Indeed, the four lowest lifetime prevalence estimates (< 10%) were in low- to middle-income countries (India, Mexico, China, South Africa). Conversely, with the exception of Brazil, the highest rates (> 18%) were in four high-income countries (France, the Netherlands, New Zealand, the USA).

**Table 2:** Prevalence (%) of DSM-IV/CIDI major depressive episodes in the 18 countries participating in the WMH surveys ^a ^

	Screen positive, mean ± SE	Lifetime prevalence, mean ± SE	Lifetime/ Screen positive, ± mean ± SE	12-month prevalence, mean ± SE	12-month/ Screen positive, mean ± SE	12-month/ lifetime, mean ± SE	Age of onset, median (IQR)^b^
**I. High-income**							

Belgium	49.4 ± 2.5	14.1 ± 1.0	28.5 ± 1.9	5.0 ± 0.5	10.0 ± 1.0	35.2(2.8	29.4 (20.9 to 41.3)

France	65.0 ± 1.7	21.0 ± 1.1	32.3 ± 1.4	5.9 ± 0.6	9.0 ± 0.9	27.9(2.6	28.4 (19.3 to 38.9)

Germany	43.1 ± 1.4	9.9 ± 0.6	23.0 ± 1.3	3.0 ± 0.3	6.9 ± 0.6	30.1 ± 2.1	27.6 (18.6 to 39.6)

Israel	45.1 ± 0.8	10.2 ± 0.5	22.6 ± 1.0	6.1 ± 0.4	13.5 ± 0.8	59.6 ± 2.3	25.5 (18.1 to 38.5)

Italy	44.9 ± 1.7	9.9 ± 0.5	22.1 ± 1.0	3.0 ± 0.2	6.7 ± 0.5	30.2 ± 1.9	27.7 (19.1 to 39.1)

Japan	29.9 ± 0.8	6.6 ± 0.5	22.2 ± 1.4	2.2 ± 0.4	7.4 ± 1.2	33.3 ± 4.2	30.1 (20.8 to 45.3)

Netherlands	53.2 ± 1.6	17.9 ± 1.0	33.6 ± 1.8	4.9 ± 0.5	9.2 ± 1.0	27.3 ± 2.6	27.2 (19.3 to 39.5)

New Zealand	61.9 ± 0.6	17.8 ± 0.4	28.7 ± 0.6	6.6 ± 0.3	10.6 ± 0.5	37.0 ± 1.5	24.2 (16.1 to 34.5)^c^

Spain	37.7 ± 1.0	10.6 ± 0.5	28.2 ± 1.2	4.0 ± 0.3	10.6 ± 0.8	37.5 ± 1.9	30.0 (19.7 to 44.3)

United States	62.0 ± 0.9	19.2 ± 0.5	30.9 ± 0.7	8.3 ± 0.3	13.3 ± 0.5	43.1 ± 1.2	22.7 (15.1 to 34.6)

Total	52.3 ± 0.4	14.6 ± 0.2	28.1 ± 0.3	5.5 ± 0.1	10.6 ± 0.2	37.7 ± 0.7	25.7 (17.3 to 37.2)

**II. Low- to middle-income**							

Sao Pâulo, Brazil	66.0 ± 1.0	18.4 ± 0.8	27.9 ± 1.1	10.4 ± 0.6	15.8 ± 0.8	56.7 ± 1.5	24.3 (17.2 to 35.8)

Colombia	58.6 ± 1.1	13.3 ± 0.6	22.6 ± 1.0	6.2 ± 0.4	10.6 ± 0.7	46.7 ± 2.6	23.5 (15.6 to 33.6)

Pondicherry, India	25.0 ± 0.9	9.0 ± 0.5	35.9 ± 1.5	4.5 ± 0.4	18.0 ± 1.4	50.0 ± 3.0	31.9 (24.5 to 42.7)

Lebanon	57.7 ± 1.8	10.9 ± 0.9	18.9 ± 1.3	5.5 ± 0.7	9.5 ± 1.2	50.0 ± 3.7	23.8 (17.5 to 32.8)

Mexico	40.6 ± 1.1	8.0 ± 0.5	19.6 ± 1.2	4.0 ± 0.3	9.8 ± 0.8	50.0 ± 2.7	23.5 (16.7 to 34.0)

Shenzen, China	54.6 ± 0.9	6.5 ± 0.4	12.0 ± 0.7	3.8 ± 0.3	6.9 ± 0.5	58.0 ± 2.6	18.8 (14.9 to 23.4)

South Africa	56.1 ± 1.3	9.8 ± 0.7	17.4 ± 1.2	4.9 ± 0.4	8.6 ± 0.8	49.6 ± 2.7	22.3 (15.8 to 33.8)

Ukraine	82.4 ± 1.1	14.6 ± 0.7	17.7 ± 0.8	8.4 ± 0.6	10.2 ± 0.7	57.8 ± 2.2	27.8 (18.7 to 39.6)

Total	54.1 ± 0.4	11.1 ± 0.2	19.8 ± 0.4	5.9 ± 0.2	10.5 ± 0.3	53.3 ± 0.9	24.0 (17.0 to 34.8)

^a^Assessed in part I sample. Prevalence for the pooled samples (developed and developing) include respondents ages 18+. Prevalence for individual countries are assessed for the total sample in the country.

^b^IQR, interquartile range.

The percentage of the screen-positive respondents who had lifetime MDE was also higher in high-income (28.1%) than in low- to middle-income (19.8%) countries, although both the lowest and the highest percentages were in low- to middle-income countries (12.0% in China vs. 35.9% in India). The ratio of the highest to lowest conditional prevalence scores in screened positives is 3.0. Among the high-income countries, these conditional prevalence estimates were relatively low (< 25%) in Germany, Italy, Israel and Japan, and higher (> 30%) in the Netherlands and USA.

We previously reported that the pooled 12-month prevalence of MDE was similar in high-income (5.5%) and low- to middle-income (5.9%) countries (*t *= 1.2, *P *= 0.25), with the specific estimates varying from 2.2% (Japan) to 8.3% (USA) in high-income countries, and from 3.8% (China) to 10.4% (Brazil) in low- to middle-income countries [11]. In the group of screen-positive respondents, the percentage with 12-month MDE was also similar for high-income (10.6%) and low- to middle-income (10.5%) countries (Table 2). The lowest rate was 6.7% (Italy) and the highest 18.0% (India). In 10 countries, these percentages were between 8 and 12%.

The ratio of the 12-month prevalence to lifetime prevalence is an indirect indicator of persistence. This ratio was significantly lower on average in surveys carried out in high-income (37.7%) than low- to middle-income (53.3%) countries (*t *= 7.5, *P *< 0.001) (Table 2). Among the high-income countries, the ratio ranged from ≤ 30% in France, Germany, Italy and the Netherlands to > 40% in the USA and Israel. Among the low- to middle-income countries, the lowest ratios were in Colombia (46.7%) and South Africa (49.6%), and the highest (57-58%) in Brazil, China and Ukraine. Consistent with these results, the 30-day prevalence of MDE was somewhat lower in high-income (mean ± SE 1.8% ± 0.1%) than in low- to middle-income (2.6 ± 0.1%) countries.

The last column of Table 2 shows that the median retrospectively reported age of onset (AOO) was similar for high-income and low- to middle-income countries (25.7 vs. 24.0, respectively) and that the interquartile ranges were largely overlapping. The 95% confidence intervals indicate that across all countries, the risk period for onset of depression ranges from the mid-late adolescence to the early 40s. In high-income countries, the earliest median AOO estimates occurred in the USA (22.7) and New Zealand (24.2), whereas the latest were in Spain (30.0) and Japan (30.1). In low- to middle-income countries, the earliest median AOO estimates were in China (18.8) and South Africa (22.3), and the latest in Ukraine (27.8) and India (31.9).

### Impairment

As expected, MDE was associated with substantial impairment in the WMH data. Moreover, the degree of impairment increased progressively with recency of MDE. (Table 3) This was true in both high and low- to middle-income countries, apart from Japan, in which the impairment level was exceptionally low. We note that the non-MDE comparison group, which has the lowest level of impairment, comprised not only healthy respondents but also respondents with other DSM-IV diagnoses (Table 3).

**Table 3:** Comparisons of functional impairment (WHO-DAS Global Scores)^a ^by recency of DSM-IV/CIDI major depressive episodes in the 18 countries participating in the WMH surveys

	Past 30 days		Past 12 months^b^		> 12 months ago		No lifetime MDE			
	
	**Mean **± **SE**	n	**Mean **± **SE**	n	**Mean **± **SE**	n	**Mean **± **SE**	n	SD^c^	*F *
**I. High-income**										

Belgium	11.3 ± 3.3+	42	7.5 ± 2.1+	71	3.5 ± 0.5	254	3.2 ± 0.7	676	8.4	3.2*

France	13.6 ± 2.6+	38	6.6 ± 1.1 +	134	4.0 ± 0.5	476	3.2 ± 0.4	788	8.4	7.9*

Germany	15.3 ± 5.4+	36	4.1 ± 1.0	73	2.4 ± 0.4	263	2.7 ± 0.3	951	7.9	2.8

Israel	21.5 ± 2.4+	82	9.7 ± 1.0+	208	6.8 ± 0.8+	211	5.0 ± 0.2	4358	12.0	24.7*

Italy	15.4 ± 2.5+	58	6.3 ± 2.0+	71	3.0 ± 0.4+	323	2.1 ± 0.2	1327	6.8	13.5*

Japan	2.9 ± 2.6	11	2.9 ± 0.8+	49	1.5 ± 0.2	125	1.0 ± 0.2	882	4.4	2.3

Netherlands	17.9 ± 2.4+	42	5.7 ± 1.1	93	5.1 ± 0.4	341	4.1 ± 0.5	618	8.8	12.5*

New Zealand	11.7 ± 1.0+	292	5.0 ± 0.5+	606	3.2 ± 0.2+	1473	2.5 ± 0.1	5064	7.6	32.9*

Spain	15.7 ± 2.2+	109	6.1 ± 1.0+	138	4.3 ± 0.6+	425	2.1 ± 0.2	1449	7.6	24.9*

United States	15.8 ± 1.2+	297	7.6 ± 0.6+	496	4.3 ± 0.2+	1002	3.0 ± 0.2	3896	9.0	54.9*

Total	15.3 ± 0.7+	1005	6.8 ± 0.3+	1942	3.9 ± 0.1+	4903	3.0 ± 0.1	20,096	8.9	149.4*

**II. Low- to middle-income**^d^										

Sao Pâulo, Brazil	12.9 ± 1.6+	260	6.8 ± 0.6+	280	4.1 ± 0.5+	413	1.8 ± 0.2	1989	7.8	44.0*

Colombia	5.1 ± 1.4+	82	3.3 ± 0.7+	194	1.8 ± 0.3+	316	0.9 ± 0.1	1789	4.2	11.1*

Pondicherry, India	2.3 ± 0.5+	71	3.2 ± 0.8+	83	1.5 ± 0.4	153	1.0 ± 0.1	1066	4.3	3.9*

Lebanon	9.3 ± 1.5+	72	3.7 ± 0.8+	71	3.4 ± 0.7+	162	1.5 ± 0.2	726	5.8	17.3*

Mexico	8.0 ± 1.8+	117	3.4 ± 0.7+	142	1.4 ± 0.3+	250	0.6 ± 0.1	1853	4.3	8.5*

Shenzen, China	3.3 ± 0.5+	101	1.7 ± 0.4+	144	0.6 ± 0.1	138	0.5 ± 0.0	2093	2.4	16.9*

Ukraine	14.8 ± 1.2+	229	10.91.1+	159	6.8 ± 1.1+	238	3.7 ± 0.3	1093	9.7	46.3*

Total	10.1 ± 0.7+	932	5.2 ± 0.3+	1073	3.1 ± 0.2+	1670	1.3 ± 0.1	10,608	6.1	112.6*

*Significantly different at the .05 level based on a 3 degree of freedom test

+Significantly different from respondents with no lifetime MDE at the +.05 level, 2-sided test

^a^WHO-DAS: World Health Organization-Disability Assessment Schedule

^b^Excludes respondents with MDE in the past 30 days

^c^SD: Standard deviation of the impairment score in the total sample

^d^Data for South Africa are not available.

For respondents with current MDE, the mean level of impairment was between approximately five (high-income) and eight (low- to middle-income) times as high as for respondents without MDE, with differences in mean scores of 12.3 (15.3 minus 3.0) in high-income countries and 8.8 (10.1 minus 1.3) in low- to middle-income countries. To put these differences into perspective, the mean differences in all high-income countries combined (15.3-3.0 = 12.3) and in all low- to middle-income countries combined (10.1-1.3 = 8.9) were both equal to 1.4 standard deviations on the impairment scale in those countries. Effect sizes such as these are large [25]. The biggest differences (greater than sevenfold) are in Italy, Spain, Brazil and Mexico and the smallest (less than fivefold) in Belgium, Israel, India and Ukraine. Respondents with MDE in the past year (but not currently) reported impairment scores between approximately twofold (high-income) and fourfold (low- to middle-income) that of the non-MDE group, although this difference was not significant in Germany or the Netherlands. The largest mean differences (greater than threefold) were in low- to middle-income countries (Mexico, Brazil, Colombia, India and China), and the smallest (approximately twofold) in four high-income countries (France, Belgium, Israel, New Zealand). In seven countries, (five high-income and two low- to middle-income), there was no significant difference in impairment between respondents with MDE prior to the past year and the non-MDE subsample. In five countries (Spain, Brazil, Colombia, Lebanon and Mexico), the MDE positive group had an approximately twofold higher level of impairment than the non-MDE group.

### The association between prevalence and impairment

As noted in the introduction, a previous cross-national WHO study carried out in primary-care waiting-room samples found that depressed respondents in countries in which the prevalence of depression was estimated to be highest reported the lowest average levels of impairment associated with their depression, whereas the highest impairment was reported by depressed respondents in countries in which the prevalence of depression was estimated to be lowest [15]. We investigated this issue in the WMH data by creating a small data file in which each survey was treated as a separate observation, and the variables were the measures of prevalence (reported in Table 2) and a measure of impairment associated with MDE (based on the results reported in Table 3). However, the impairment scores differed from those in Table 3 in that they represented the difference in mean impairment scores of respondents with 12-month MDE compared with those with no lifetime history of MDE in the survey. This difference was taken to represent the effect of recent MDE as assessed by the CIDI on impairment in the survey.

Unlike the earlier primary-care study, we found that the association between prevalence and impairment was positive. (Table 4 Table 5) This was true not only in the total sample of all countries (*r *= 0.48) but also when we looked separately at high-income (*r *= 0.34) and low- to middle-income (*r *= 0.80) countries. In addition, when we deconstructed these associations into correlations of impairment with the two components of prevalence (the percentage of respondents endorsing an MDE stem question and the conditional prevalence estimate of MDE of screen-positives), we found that the first correlation was considerably stronger than the second in both the total sample of countries (*r *= 0.45, 0.11) and in low- to middle-income countries (*r *= 0.76, 0.04), whereas the first correlation was stronger than the second in high-income countries (*r *= 0.17, 0.45).

**Table 4:** Associations of demographic characteristics with 12-month DSM-IV/CIDI major depressive episode in high-income countries (bivariate analyses)^a^

Parameter	Total, OR (95% CI)	Belgium, OR (95% CI)	France, OR (95% CI)	Germany, OR (95% CI)	Israel, OR (95% CI)	Italy, OR (95% CI)	Japan, OR (95% CI)	Netherlands, OR (95% CI)	New Zealand, OR (95% CI)	Spain, OR (95% CI)	USA, OR (95% CI)
**Gender**											
Women	1.8 (1.6 to 2.0)*	1.6 (0.9 to 2.8)	1.7 (1.2 to 2.5)*	1.7 (1.0 to 3.0)	1.6 (1.2 to 2.1)*	2.5 (1.6 to 3.8)*	2.3 (1.4 to 4.0)*	2.3 (1.5 to 3.5)*	1.7 (1.4 to 2.1)*	2.7 (1.9 to 3.8)*	1.7 (1.4 to 2.1)*
Men	1.0	1.0	1.0	1.0	1.0	1.0	1.0	1.0	1.0	1.0	1.0
**Age**											
18-34	2.7 (2.3 to 3.1)*	2.6 (0.9 to 7.7)	3.5 (1.7 to 7.4)*	3.8 (1.6 to 9.2)*	1.1 (0.7 to 1.6)	0.8 (0.5 to 1.5)	4.8 (2.3 to 10.0)*	2.6 (1.2 to 5.7)*	5.5 (3.9 to 7.8)*	1.0 (0.6 to 1.7)	4.3 (3.1 to 6.0)*
35-49	2.2 (1.9 to 2.6)*	2.2 (1.0 to 4.8)*	2.5 (1.2 to 5.3)*	2.3 (1.0 to 5.5)	1.0 (0.6 to 1.4)	0.7 (0.4 to 1.3)	2.7 (1.3 to 5.6)*	2.5 (1.2 to 5.4)*	4.4 (3.2 to 6.2)*	1.1 (0.7 to 1.6)	3.9 (2.7 to 5.5)*
50-64	2.0 (1.7 to 2.3)*	2.5 (1.1 to 5.6)*	2.3 (1.1 to 4.7)*	2.4 (1.0 to 5.5)*	1.0 (0.7 to 1.6)	1.2 (0.7 to 2.1)	2.4 (1.2 to 4.8)*	1.9 (0.9 to 3.8)*	2.9 (2.0 to 4.1)*	1.6 (1.1 to 2.3)*	3.1 (2.1 to 4.5)*
65+	1.0	1.0	1.0	1.0	1.0	1.0	1.0	1.0	1.0	1.0	1.0
**Marital status**^b^											
Separated	3.6 (2.9 to 4.6)*	7.3 (1.8 to 29.7)*	6.2 (1.8 to 21.3)*	-	2.6 (1.0 to 6.8)	2.8 (1.1 to 7.5)*	10.8 (2.1 to 55.6)*	-	3.4 (2.4 to 4.8)*	3.2 (1.3 to 7.7)*	4.0 (2.7 to 6.0)*
Divorced	2.1 (1.8 to 2.5)*	1.9 (0.7 to 5.3)	1.1 (0.5 to 2.5)	3.1 (1.4 to 7.1)*	2.2 (1.5 to 3.4)*	0.6 (0.1 to 5.0)	5.1 (2.1 to 12.6)*	2.7 (1.5 to 4.9)*	2.8 (2.0 to 3.8)*	3.3 (1.2 to 8.9)*	1.7 (1.3 to 2.3)*
Widowed	1.4 (1.2 to 1.7)*	1.4 (0.6 to 3.4)	1.5 (0.7 to 3.2)	2.3 (1.2 to 4.5)*	2.1 (1.4 to 3.3)*	1.5 (0.9 to 2.7)	0.9 (0.4 to 2.1)	0.8 (0.3 to 2.2)	1.3 (0.9 to 1.8)	1.4 (0.9 to 2.2)	1.2 (0.8 to 1.9)
Never married	1.8 (1.6 to 2.0)*	1.3 (0.6 to 2.9)	2.0 (1.2 to 3.5)*	2.6 (1.6 to 4.2)*	1.4 (1.0 to 1.9)*	1.5 (1.0 to 2.2)	3.1 (1.6 to 5.7)*	1.8 (1.0 to 3.4)	2.3 (1.8 to 3.0)*	0.9 (0.6 to 1.4)	1.8 (1.5 to 2.1)*
Currently married	1.0	1.0	1.0	1.0	1.0	1.0	1.0	1.0	1.0	1.0	1.0
**Living arrangements**											
Alone	1.8 (1.6 to 2.0)*	1.3 (0.6 to 2.7)	1.4 (0.9 to 2.3)	2.5 (1.6 to 3.9)*	2.1 (1.5 to 2.9)*	1.7 (1.1 to 2.8)*	2.9 (1.2 to 6.8)*	1.6 (1.1 to 2.4)*	1.8 (1.4 to 2.3)*	1.0 (0.7 to 1.6)	1.7 (1.4 to 2.2)*
With others	1.9 (1.7 to 2.2)*	1.5 (0.8 to 2.8)	2.2 (1.4 to 3.7)*	2.9 (1.6 to 5.1)*	1.6 (1.2 to 2.1)*	1.4 (0.9 to 2.1)	3.0 (1.6 to 5.6)*	1.9 (0.8 to 4.3)	2.5 (2.0 to 3.1)*	1.2 (0.8 to 1.7)	1.8 (1.5 to 2.1)*
With spouse	1.0	1.0	1.0	1.0	1.0	1.0	1.0	1.0	1.0	1.0	1.0
**Income**											
Low	1.7 (1.5 to 2.0)*	1.3 (0.7 to 2.6)	2.4 (1.2 to 4.6)*	2.7 (1.3 to 5.6)*	1.1 (0.7 to 1.7)	1.3 (0.6 to 2.9)	0.5 (0.2 to 1.4)	1.1 (0.6 to 1.9)	2.2 (1.6 to 3.0)*	1.0 (0.5 to 2.0)	2.1 (1.5 to 2.8)*
Low average	1.3 (1.1 to 1.5)*	1.1 (0.5 to 2.4)	1.4 (0.7 to 2.6)	1.6 (0.7 to 3.5)	0.9 (0.6 to 1.3)	1.2 (0.6 to 2.3)	0.5 (0.2 to 1.2)	0.8 (0.4 to 1.8)	1.5 (1.2 to 2.0)*	1.1 (0.7 to 1.8)	1.4 (1.0 to 1.8)*
High average	1.1 (0.9 to 1.2)	0.9 (0.4 to 2.0)	1.3 (0.7 to 2.3)	1.6 (0.9 to 3.1)	0.8 (0.6 to 1.1)	0.9 (0.5 to 1.5)	0.5 (0.2 to 1.3)	0.8 (0.5 to 1.6)	1.3 (1.0 to 1.7)	1.0 (0.6 to 1.5)	1.1 (0.8 to 1.5)
High	1.0	1.0	1.0	1.0	1.0	1.0	1.0	1.0	1.0	1.0	1.0
**Education**											
Low	1.0 (0.9 to 1.2)	1.4 (0.8 to 2.5)	-	1.0 (0.2 to 4.3)	1.5 (1.0 to 2.2)*	1.5 (0.9 to 2.5)	0.2 (0.1 to 0.6)*	1.1 (0.6 to 1.9)	0.9 (0.7 to 1.2)	1.2 (0.7 to 1.9)	1.4 (1.1 to 1.8)*
Low average	1.1 (1.0 to 1.3)	0.9 (0.4 to 2.2)	-	1.6 (0.4 to 5.8)	1.3 (0.9 to 1.8)	0.8 (0.4 to 1.4)	0.7 (0.4 to 1.4)	1.3 (0.7 to 2.6)	1.1 (0.8 to 1.4)	0.8 (0.5 to 1.3)	1.2 (0.9 to 1.5)
High average	1.1 (0.9 to 1.3)	1.8 (0.8 to 3.8)	-	1.1 (0.2 to 5.2)	1.0 (0.7 to 1.6)	0.9 (0.5 to 1.5)	0.7 (0.3 to 1.6)	1.5 (0.7 to 3.2)	0.9 (0.7 to 1.1)	1.0 (0.7 to 1.6)	1.4 (1.0 to 1.8)*
High	1.0	1.0	-	1.0	1.0	1.0	1.0	1.0	1.0	1.0	1.0

*Significant at the 0.05 level, two-sided test.

^a^All models were bivariate models with the sociodemographic factors as predictors and 12-month MDE as the response variable. The models for total (first column) control for countries. The models for income were estimated in part II samples, whereas all other models were estimated in part I samples.

^b^In some countries, people were categorized as separated/widowed/divorced because they were known to have married previously but not any longer, but the specific category was unknown. These cases were dropped from the model using marriage as the predictor. Specifically, there was one such case in Japan, two cases in the USA, one case in New Zealand, and ninety-one in the ESEMeD countries (France, Germany, Italy, Netherlands, Spain, Belgium).

**Table 5:** Associations of demographic characteristics with 12-month DSM-IV/CIDI major depressive episode in low- to middle-income countries: bivariate analyses)^a^

	Total, OR (95% CI)	Brazil, OR (95% CI)	Colombia, OR (95% CI)	India, OR (95% CI)	Lebanon, OR (95% CI)	Mexico, OR (95% CI)	China, OR (95% CI)	South Africa, OR (95% CI)	Ukraine, OR (95% CI)
**Gender**									
Women	2.1 (1.8 to 2.3)*	2.6 (1.9 to 3.5)*	1.9 (1.4 to 2.7)*	1.9 (1.3 to 2.7)*	2.1 (1.3 to 3.4)*	2.1 (1.5 to 2.9)*	1.2 (0.8 to 1.7)	2.2 (1.5 to 3.2)*	2.5 (2.0 to 3.0)*
Men	1.0	1.0	1.0	1.0	1.0	1.0	1.0	1.0	1.0
**Age**									
18-34	0.9 (0.8 to 1.1)	3.0 (1.6 to 5.7)*	4.9 (0.9 to 28.3)	0.4 (0.2 to 1.0)*	1.7 (0.8 to 3.7)	0.4 (0.2 to 1.2)	2.8 (0.8 to 9.4)	1.3 (0.6 to 2.6)	0.4 (0.3 to 0.6)*
35-49	1.0 (0.8 to 1.2)	3.3 (1.7 to 6.5)*	3.9 (0.7 to 23.1)	1.2 (0.6 to 2.5)	2.2 (1.0 to 5.2)	0.5 (0.2 to 1.3)	1.6 (0.5 to 5.3)	1.6 (0.8 to 3.2)	0.5 (0.3 to 0.7)*
50-64	1.0 (0.8 to 1.3)	2.5 (1.4 to 4.5)*	3.4 (0.6 to 20.1)	1.5 (0.7 to 3.2)	1.9 (0.9 to 4.1)	0.5 (0.2 to 1.5)	0.8 (0.2 to 3.3)	1.7 (0.8 to 3.6)	0.7 (0.5 to 1.1)
65+	1.0	1.0	1.0	1.0	1.0	1.0	1.0	1.0	1.0
**Marital status^b^**									
Separated	1.7 (1.3 to 2.2)*	1.6 (1.1 to 2.3)*	0.9 (0.6 to 1.6)	8.2 (2.2 to 30.6)*	19.3 (5.0 to 74.4)*	1.9 (1.0 to 3.6)*	-	2.7 (0.7 to 9.6)	6.6 (1.1 to 38.0)*
Divorced	3.0 (2.4 to 3.9)*	3.0 (1.9 to 4.9)*	1.2 (0.3 to 4.3)	-	0.8 (0.2 to 4.2)	1.2 (0.4 to 3.8)	6.2 (2.2 to 17.3)*	2.1 (1.3 to 3.5)*	4.2 (2.9 to 6.2)*
Widowed	2.7 (2.2 to 3.2)*	1.1 (0.7 to 1.9)	1.6 (0.9 to 2.9)	2.2 (1.5 to 3.2)*	1.4 (0.6 to 3.6)	2.7 (1.5 to 5.0)*	4.1 (0.8 to 20.7)	2.3 (1.3 to 4.0)*	8.0 (5.3 to 12.0)*
Never married	1.0 (0.9 to 1.1)	1.0 (0.7 to 1.3)	1.3 (0.9 to 1.8)	0.3 (0.1 to 0.6)*	1.3 (0.8 to 2.0)	0.8 (0.5 to 1.3)	1.4 (1.0 to 1.9)	0.7 (0.5 to 1.0)*	0.8 (0.5 to 1.3)
Currently married	1.0	1.0	1.0	1.0	1.0	1.0	1.0	1.0	1.0
**Living arrangements**									
Alone	1.6 (1.4 to 1.9)*	1.3 (0.8 to 2.0)	0.6 (0.3 to 1.2)	1.2 (0.6 to 2.5)	1.3 (0.7 to 2.5)	1.0 (0.5 to 1.9)	1.6 (1.1 to 2.3)*	1.2 (0.8 to 1.9)	2.5 (1.9 to 3.3)*
With others	1.2 (1.1 to 1.3)*	1.2 (1.0 to 1.5)	1.3 (0.9 to 1.8)	0.6 (0.4 to 0.9)*	1.4 (0.8 to 2.2)	1.1 (0.8 to 1.6)	0.9 (0.4 to 2.1)	0.9 (0.6 to 1.2)	1.5 (1.1 to 2.0)*
With spouse	1.0	1.0	1.0	1.0	1.0	1.0	1.0	1.0	1.0
**Income**									
Low	1.1 (0.9 to 1.3)	1.0 (0.7 to 1.5)	1.4 (0.8 to 2.3)	1.7 (0.9 to 3.2)	1.0 (0.5 to 2.3)	2.1 (1.4 to 3.2)*	0.7 (0.4 to 1.2)	0.9 (0.6 to 1.4)	0.7 (0.4 to 1.3)
Low average	1.1 (0.9 to 1.3)	1.1 (0.8 to 1.6)	1.5 (0.9 to 2.5)	2.1 (1.2 to 3.8)*	1.1 (0.5 to 2.5)	1.5 (1.0 to 2.3)	0.6 (0.3 to 1.0)	0.9 (0.5 to 1.4)	0.8 (0.4 to 1.4)
High average	1.0 (0.8 to 1.2)	1.2 (0.8 to 1.8)	0.8 (0.5 to 1.3)	2.0 (1.2 to 3.5)*	1.3 (0.6 to 2.7)	1.4 (0.8 to 2.2)	0.5 (0.3 to 0.8)*	0.5 (0.3 to 1.2)	0.9 (0.5 to 1.7)
High	1.0	1.0	1.0	1.0	1.0	1.0	1.0	1.0	1.0
**Education**									
Low	1.1 (1.0 to 1.4)	0.7 (0.5 to 1.1)	0.9 (0.6 to 1.4)	14.1 (3.4 to 58.9)*	1.2 (0.7 to 2.1)	2.1 (1.3 to 3.2)*	0.2 (0.1 to 0.6)*	0.5 (0.2 to 1.5)	2.3 (1.4 to 3.8)*
Low average	1.1 (0.9 to 1.3)	1.1 (0.8 to 1.6)	1.2 (0.7 to 1.9)	1.0 (0.1 to 12.5)	1.2 (0.6 to 2.4)	1.6 (1.0 to 2.5)	0.5 (0.3 to 0.7)*	2.1 (1.1 to 4.1)*	1.3 (0.9 to 1.8)
High average	0.9 (0.8 to 1.1)	0.9 (0.7 to 1.2)	0.9 (0.5 to 1.5)	3.9 (0.6 to 27.0)	1.3 (0.7 to 2.4)	1.2 (0.7 to 1.9)	0.8 (0.5 to 1.2)	1.0 (0.5 to 1.9)	1.0 (0.6 to 1.5)
High	1.0	1.0	1.0	1.0	1.0	1.0	1.0	1.0	1.0

*Significant at the 0.05 level, two-sided *t*-test

^a^All models were bivariate models with the sociodemographic factors as predictors and 12-month MDE as the response variable. The models for total (first column) control for countries. The models for income were estimated in part II samples, whereas all other models were estimated in part I samples.

^b^In some countries, people were categorized as separated/widowed/divorced because they were known to have married previously but not anymore, but the specific category was unknown. These cases were dropped from the model using marriage as the predictor. There were 48 cases in India, 35 in Brazil, 664 in Ukraine and 1 case in South Africa.

### Sociodemographic factors

Tables 4 and 5 show the bivariate associations of the sociodemographic characteristics with 12-month MDE (tables showing the country-specific distributions of the demographic variables and 12-month prevalences of MDE are available upon request). Consistent with previous epidemiologic studies, women were on average twice as likely as men to be classified as having MDE. This difference was significant in 15 of the eighteen countries, and even in the three exceptions (Belgium, Germany and China), women had higher rates than men. In the developed countries, the significant odds ratios ranged from 1.6 in Israel to 2.7 in Spain, and in the developing countries, they ranged from 1.9 in India and Colombia to 2.6 in Brazil. The association between gender and MDE did not differ significantly between high-income and low- to middle-income countries (χ^2^_1 _= 2.3, *P *= 0.13).

The associations between age group and MDE varied considerably between countries. In two high-income and five low- to middle-income countries, there were no significant associations. In six high-income countries and in Brazil, respondents in the youngest age group (18 to 34) were 3 to 5.5 times as likely to have MDE as those in the oldest age group (65+), but in India and Ukraine, young age was associated with low risk. The 35 to 49 year age group was also at increased risk for MDE, especially in New Zealand (OR = 4.4), the USA (OR = 3.9) and Brazil (OR = 3.3); in Ukraine, however, they had a significantly lower risk than those in the oldest age group. Mid-life (ages 50 to 64), encompasses a period of transition from work to retirement in many countries. Compared with respondents age 65+, participants in this group had an increased risk of MDE in eight high-income countries and Brazil, with ORs ranging from 1.6 (Spain) to 3.1 (USA). Overall, the association between age and MDE was significantly stronger in high-income than low- to middle-income countries (χ^2^_3 _= 67.1, p < .001).

Marital status was a consistently significant correlate of MDE. Being separated was associated with increased risk of MDE in twelve countries, with odds ratios varying from < 4.0 in five countries to > 8.0 in India (OR = 8.2), Japan (OR = 10.8) and Lebanon (OR = 19.3). Being divorced was associated with MDE in seven of the ten developed and four of the eight developing countries, with unusually high ORs in Japan (OR = 5.1), China (OR = 6.2) and Ukraine (OR = 4.2). Being widowed was less consistently and more modestly associated with MDE with the exception of Ukraine, where widows were eight times as likely as married men and women to have MDE. In the high-income countries, there was a significantly increased OR of MDE among the never married. However, India and South Africa were the only two low- to middle-income samples with significant ORs, and in these countries never being married was associated with low risk. Overall, the association between marital status and MDE differed significantly between high and low- to middle-income countries (χ^2^_3 _= 124.4, *P *< 0.001), due to stronger associations of being separated and never married with depression in high-income countries, and stronger associations of being divorced and widowed with depression in low- to middle-income countries. In contrast to marital status, living arrangements *per se *were more modestly associated with MDE. This association was significant in eight of the high-income countries and in Ukraine and China, with the overall difference in the association between high and low- to middle-income countries significant (χ^2^_2 _= 39.0, *P *< 0.001) due to a higher OR between being unmarried but living with others in high than in low- to middle-income countries.

The poorest respondents in France, Germany, New Zealand and the USA had an approximately twofold increased odds of MDE compared with those in the highest income group. In the low- to middle-income countries, in comparison, income was not significantly related to MDE. This stronger association between income and MDE in higher-income countries was significant overall (χ^2^_3 _= 19.3, *P *< 0.001). Similarly, among the non-Asian countries, low education was significantly associated with MDE only in Israel, the USA, Mexico and Ukraine. The findings for the Asian countries were more complex. In India, respondents with the lowest education were 14 times as likely to have MDE as those with the highest education. In Japan and China, the reverse pattern was found, with the least educated having the lowest risk of MDE. The association between education and MDE overall did not differ significantly between high and low- to middle-income countries (χ^2^_3 _= 6.2, *P *= 0.10).

## Discussion

Consistent with previous cross-national reports, the WMH MDE prevalence estimates varied considerably between countries, with the highest prevalence estimates found in some of the wealthiest countries in the world. However, contrary to our initial expectation, we found no evidence that this wide cross-national variation was due as much to cross-national differences in endorsing diagnostic stem questions as to conditional prevalence of MDE among respondents who endorsed a diagnostic stem. The ratio of the highest to lowest screen-positive rates across countries (3.3) was very similar to the ratio of the highest to lowest conditional prevalence rates among screen-positives (3.0). As expected, we also found that MDE was associated with substantial impairment. However, contrary to our initial expectation, we did not find that cross-national differences in prevalence estimates were inversely related to differences in average level of impairment associated with depression; indeed, the opposite pattern was found.

Taken together, these results argue against the suggestion that the wide cross-national variation in depression prevalence estimates in the WMH surveys and previous epidemiologic studies is due to the threshold for defining clinically significant depression in standard diagnostic interviews differing across countries. If that were the case, we would expect that the cases of depression detected in countries with the lowest estimated prevalence of depression would be the most severe cases, resulting in high impairment rates among these cases, whereas the opposite would be true in countries with the highest estimated prevalence of depression. Furthermore, we would expect that reports of core depressive symptoms would be more similar across countries than estimates of disorder prevalence. Neither of these expectations was borne out in the WMH data. A question can be raised by our results regarding why the associations between prevalence and impairment were so different from those reported in the earlier WHO study [15]; however, it is important to bear in mind that this earlier study was based on primary-care samples, for which selection bias regarding seeking help on the basis of either distress or impairment might induce a more negative association between these two variables than exists in the population. The WMH surveys, by contrast, are based on general population samples, for which the selection bias issues that occur in treatment samples do not arise.

Although these results add indirect support to a substantive interpretation of the cross-national differences in MDE found here, they shed no light on why these differences exist. Differences in stress exposure, in reactivity to stress, and in endogenous depression unrelated to environmental provoking factors are all possibilities. On one level, it seems counterintuitive that people in high-income countries should experience more stress than those in low- to middle-income countries. However, it has been suggested that depression is to some extent an illness of affluence [26]. A related argument is that income inequality, which is for the most part greater in high than low- to middle-income countries, promotes a wide variety of chronic conditions that includes depression [27]. Further analyses of the WMH data might be able to shed some light on these perspectives; however such an analysis was beyond the scope of the current report, which focused on the evaluation of a more methodological interpretation of the observed cross-national differences in depression prevalence estimates.

In considering a substantive interpretation of our findings, it is noteworthy that although lifetime prevalence estimates were found to be significantly higher in high than low- to middle-income countries overall, no significant difference in 12-month prevalence was found. The ratio of 12-month to lifetime prevalence estimates, furthermore, was significantly higher in low- to middle-income than in high-income countries. It might be that these results reflect genuinely lower lifetime prevalence but higher persistence of depression in low- to middle-income than high-income countries, but another plausible and more parsimonious explanation is that error in recall of previous lifetime episodes is higher in low- to middle-income than high-income countries. Longitudinal data collection would be required to document such a difference rigorously [28,29]. Although such data do not exist in all WMH series, it is important to recognize this possibility of cross-national variation in recall error before launching an extensive investigation of substantive explanations. It might be that a fruitful focus of subsequent WMH analysis would be on the youngest respondents, where lifetime recall error might be least pronounced. Alternatively, it might be that the investigation of cross-national differences in lifetime prevalence should be abandoned in favor of a focus on recent prevalence in recognition of the plausibility of significant cross-national variation in recall error of lifetime prevalence.

Another implication of the methodological limitation of the WMH surveys being all cross-sectional is that it made it impossible to determine the temporal direction of the associations examined between depression and the sociodemographic variables. This means that even though variables such as education and marital status were considered predictors of depression, they might actually have been consequences or involved in reciprocal causal relationships with depression. However, within the context of that limitation, the sociodemographic patterns reported here are broadly consistent with those found in previous community epidemiologic surveys of depression [2,5,7,9,13], adding to confidence in the generalizability of the WMH finding.

The results reported here have several other limitations, relating more generally to the WMH findings [30]. Some of the most important of these issues involve sampling. The response rates varied widely. Although the response rates did not appear to be related to depression prevalence, it is possible that in some settings, particularly those where treatment is unavailable, the most depressed people were unable to participate. Some surveys only included metropolitan areas, whereas others involved national samples. This too may have affected estimates of cross-national variation in prevalence. In addition, the surveys did not include institutionalized patients, people in jails and prisons, people in the military, people who were too intoxicated to be interviewed, or people with severe cognitive or physical disabilities. The samples also reflected survivor bias, which could be of considerable importance for understanding differences between high-income and low- to middle-income countries, given the gap in life expectancy of 10 to 15 years between people in developed and developing countries [31]. Thus, the rates reported here provide conservative estimates of MDE prevalence. A final noteworthy sample bias is that South Africa was the only African country included in this report [32] even though the WMH survey was also conducted in Nigeria [33]. Nigeria was excluded because of the extremely low prevalence of MDE (3.1% lifetime; 1.1% 12-month) and other disorders. These low prevalence estimates raise questions about the willingness of respondents in the Nigerian survey to disclose symptoms to strangers or lay interviewers, and the appropriateness of the CIDI structure for that setting [34]. They also reduced our statistical power to examine the associations of depression considered in the Nigerian data. A similar experience may have occurred in another African population-based survey using the CIDI that was not part of the WMH series. That survey, carried out in Addis Ababa, also found low rates of affective disorders [35]. Given the high level of exposure to trauma in extremely poor countries such as these [36], research is urgently needed to determine the best approaches to study the prevalence of mental disorders in these settings.

The measure of MDE also had inherent limitations. The structure of the CIDI, including the choice of stem questions in the screening section, may have led to underestimates of depression in some settings. As noted above in the section on measurement, the interview translation, back-translation and harmonization process in the WMH surveys included customization within countries of the terms used to describe the core symptoms of depression (that is, sadness, depression, loss of interest) based on clinical experiences of local collaborators and the results of pilot studies [19]. However, no attempt was made to develop distinct cut-off points in the CIDI diagnostic algorithms for different countries or to go beyond the DSM-IV criteria to develop distinct criteria for different countries that might have increased our ability to detect depression or depression-equivalents. It is noteworthy that in the countries for which we carried out blinded clinical reappraisal interviews with subsamples of WMH respondents, we found no evidence for systematic bias in the diagnostic threshold for depression [18], but clinical reappraisal interviews were not carried out in all WMH countries, and it is conceivable that such studies would have found systematic differences in the ability of the CIDI to detect clinical depression across countries.

Despite these limitations, the WMH data provide useful new information about the epidemiology of MDE. We found wide variation not only in the prevalence of MDE but also in the proportion of people who endorsed diagnostic stem questions for MDE, a pattern that has seldom been examined in previous epidemiologic studies [37]. We found cross-national consistency, by contrast, in the impairment associated with MDE. This association has to our knowledge never been considered previously in cross-national community epidemiologic surveys. Our results confirm the public-health importance of major depression as a commonly occurring and seriously impairing condition with a generally early AOO and persistent course in a wide range of countries. In addition, we replicated previous findings on the sociodemographic correlates of MDE. We also documented an intriguing opposite-sign pattern of differences between high and low- to middle-income countries in estimates of lifetime prevalence and persistence of MDE, which might be due to differences in recall error. Future research on cross-national differences in depression needs to take this pattern into consideration, and to develop a workable strategy to deal with the possibility of differential recall error as a plausible contributor to cross-national differences in prevalence estimates.

## Competing interests

RCK has been a consultant for AstraZeneca, Analysis Group, Bristol-Myers Squibb, Cerner-Galt Associates, Eli Lilly & Company, GlaxoSmithKline Inc., HealthCore Inc., Health Dialog, Integrated Benefits Institute, John Snow Inc., Kaiser Permanente, Matria Inc., Mensante, Merck & Co, Inc., Ortho-McNeil Janssen Scientific Affairs, Pfizer Inc., Primary Care Network, Research Triangle Institute, Sanofi-Aventis Groupe, Shire USA Inc., SRA International, Inc., Takeda Global Research & Development, Transcept Pharmaceuticals Inc. and Wyeth-Ayerst; has served on advisory boards for Appliance Computing II, Eli Lilly & Company, Mindsite, Ortho-McNeil Janssen Scientific Affairs, Plus One Health Management and Wyeth-Ayerst; and has had research support for his epidemiological studies from Analysis Group Inc., Bristol-Myers Squibb, Eli Lilly & Company, EPI-Q, GlaxoSmithKline, Johnson & Johnson Pharmaceuticals, Ortho-McNeil Janssen Scientific Affairs., Pfizer Inc., Sanofi-Aventis Groupe and Shire USA, Inc. The remaining authors report no competing interests.

## Authors' contributions

All authors were involved in data collection in their individual countries and participation in the design of the study for this report. EB, NAS, IH and RCK carried out the data analyses. All authors made critical revisions and approved the final manuscript.

## Pre-publication history

The pre-publication history for this paper can be accessed here:

http://www.biomedcentral.com/1741-7015/9/90/prepub
